# Reporting guidelines for population pharmacokinetic analyses

**DOI:** 10.1007/s10928-015-9417-1

**Published:** 2015-04-30

**Authors:** Kevin Dykstra, Nitin Mehrotra, Christoffer Wenzel Tornøe, Helen Kastrissios, Bela Patel, Nidal Al-Huniti, Pravin Jadhav, Yaning Wang, Wonkyung Byon

**Affiliations:** qPharmetra LLC, Andover, MA USA; Division of Pharmacometrics, US Food and Drug Administration, Silver Spring, MD USA; Clinical Reporting, Novo Nordisk, Copenhagen, Denmark; Pharsight Consulting Services, Certara, St. Louis, MO USA; Clinical Pharmacology, Quantitative Sciences, GlaxoSmithKline, King of Prussia, PA USA; Quantitative Clinical Pharmacology, AstraZeneca, Waltham, MA USA; Quantitative Pharmacology and Pharmacometrics, Merck and Co., Whitehouse Station, NJ USA; Global Innovative Pharma Business Clinical Pharmacology, Pfizer Inc., Groton, CT USA

**Keywords:** Pharmacometrics, Population pharmacokinetics, PK reporting, Regulatory submission, Best practices

## Abstract

The purpose of this work was to develop a consolidated set of guiding principles for reporting of population pharmacokinetic (PK) analyses based on input from a survey of practitioners as well as discussions between industry, consulting and regulatory scientists. The survey found that identification of population covariate effects on drug exposure and support for dose selection (where population PK frequently serves as preparatory analysis to exposure–response modeling) are the main areas of influence for population PK analysis. The proposed guidelines consider two main purposes of population PK reports (1) to present key analysis findings and their impact on drug development decisions, and (2) as documentation of the analysis methods for the dual purpose of enabling review of the analysis and facilitating future use of the models. This work also identified two main audiences for the reports: (1) a technically competent group responsible for in-depth review of the data, methodology, and results, and (2) a scientifically literate, but not technically adept group, whose main interest is in the implications of the analysis for the broader drug development program. We recommend a generalized question-based approach with six questions that need to be addressed throughout the report. We recommend eight sections (Synopsis, Introduction, Data, Methods, Results, Discussion, Conclusions, Appendix) with suggestions for the target audience and level of detail for each section. A section providing general expectations regarding population PK reporting from a regulatory perspective is also included. We consider this an important step towards industrialization of the field of pharmacometrics such that non-technical audience also understands the role of pharmacometrics analyses in decision making. Population PK reports were chosen as representative reports to derive these recommendations; however, the guiding principles presented here are applicable for all pharmacometric reports including PKPD and simulation reports.

**Electronic supplementary material:**

The online version of this article (doi:10.1007/s10928-015-9417-1) contains supplementary material, which is available to authorized users.

## Introduction

Since the early development of software for conducting non-linear mixed effects modelling in the late 1970s by Sheiner and Beal [1], population pharmacokinetics (PK) has evolved into one of the core data analysis methods utilized in drug development. Population PK is useful because it allows systematic integration of data that may have been collected in different ways from a variety of sources, and because it facilitates characterization of the effects of intrinsic patient characteristics or extrinsic treatment or study design attributes on drug exposure into a single, cohesive mathematical framework. Further it allows the simultaneous characterization of main, expected effects together with inter- and intra-subject variability on pharmacokinetic behaviour. These models may be used to simulate expected exposure metrics (e.g. AUC, C_max_, C_min_, C_avg_, etc.) under new conditions such as alternative dosing regimens or to derive individual PK parameter/exposure metrics that can be used in subsequent assessment of exposure–response relationships. As the utility and impact of pharmacometrics generally, and population PK specifically, has grown, so has the need for consistency in the ways in which these analyses are conducted and reported.

In this article, we present a consolidated set of guiding principles for reporting of population pharmacokinetic analyses based on survey input as well as discussions between industry, consulting and regulatory scientists. Our goal was to form pragmatic recommendations in order to (1) enhance communication of analysis findings; (2) broaden the impact of population PK reports; and (3) facilitate efficient report development and review.

### Motivation

In general, a population PK report serves two critical functions. The first is to communicate the key findings of the analysis in a language that is understandable to all stakeholders. In many organizations, stakeholders frequently include knowledgeable readers from varied fields of expertise in addition to experienced pharmacometricians. The report should describe the analysis objectives and intended application, clinical relevance of the findings, and place the results in the appropriate drug development context.

The second major purpose of the population PK report is to provide detailed documentation of analysis methods and conduct, enabling the work to be reproduced if needed, or to facilitate review (internal and/or regulatory). The document needs to include description of the data used and reasoning by which population PK models were developed. Further, the report needs to include sufficient evidence that the model adequately describes the data, and, thus, can be reasonably used to, for example, predict future exposure in the population(s) of interest. This documentation needs to be sufficiently detailed to allow a reader to reproduce the results described in the document. This function of the report is critical not only for formal reviewers, but also for pharmacometricians who may need to expand on or otherwise refer to a given analysis months or years after it was originally performed.

In the authors’ experience, there is substantial variation in the content and formatting of population PK reports generated by different organizations and even among pharmacometricians within a single organization, and less than clear consensus as to what parts of a given analysis should be highlighted to maximize the usefulness of the results. Frequent shortcomings of population PK reports include:

- Analysis objectives and application not clearly stated
- Important findings and their relevance to analysis objectives are overshadowed by technical detail
- Many graphical and tabular data displays are used to examine and qualify models, but provide little insight into the impact of the key findings

It is anticipated that wide implementation of practical reporting guidelines should have the overriding benefit of facilitating clear communication of the analysis results to all interested stakeholders. The report should highlight analysis objectives, demonstrate how the analysis met those objectives, facilitate ready access to the results and describe their clinical impact, and enhance readability of key sections to a generally knowledgeable audience. Additionally, implementation of these guidelines should increase the efficiency of reviewing these reports by enhancing the consistency of formatting and content in the documents, allowing the reader to easily locate the information critical to assessing the validity and strength of the findings. Use of standardized reporting formats should enable drug development organizations to produce these documents more efficiently, utilizing automation where appropriate, and eliminating needlessly repetitive description of standardized methods. Finally, application of reporting standards constitutes an important step toward the industrialization of pharmacometrics [2].

### Previous work

Several authors have touched on the methodology for population PK conduct and reporting over the past decade. There have been many software and methodological advances since the first FDA guidance on popPK was finalized in 1999 [3]. Wade [4] published guidelines specific to the Swedish Medical Products Agency, and a guidance for population PK was formalized by EMA in 2007 [5]. Notable about these recommendations is an emphasis on very detailed description of the model and model qualification and less emphasis on highlighting the purpose and application of the models. More recently, best practices utilized in two industry-based pharmacometrics groups have been published [6, 7] focusing on guidelines for general population PK modeling within single companies and Jamsen et al. [8] published a journal’s perspective on reporting population PK studies.

### Approach

This work was carried out under the auspices of the Model-Based Drug Development (MBDD) Consortium, a working group comprised of representatives from American Society of Clinical Pharmacology and Therapeutics (ASCPT), American College of Clinical Pharmacology (ACCP), American Association of Pharmaceutical Sciences (AAPS), and the International Society of Pharmacometrics (ISoP). Our group included representatives from industry, pharmacometric consultancies, and the FDA.^1^

In developing our recommendations, we initially conducted a survey of population PK reporting practices among interested scientific society members in order to identify preferences and practices that are common among practitioners. These results formed the starting point for development of the guiding principles. The form and use of population PK reports were further discussed in order to highlight aspects of the reporting process and report content that function well, and those that did not. Topics included current (and aspirational) target audiences for the reports, examination of the functional purpose of these documents, degree to which some practices are sufficiently standardized to make it less critical to include their exhaustive discussion in the report, and depth of detail in the documentation that allows an analysis to be qualified as fit for purpose.

The survey results and emerging recommendations were presented at each of the MBDD member society national meetings in order to encourage comments from the broader pharmacometrics community, and these suggestions are incorporated here where appropriate. We especially wanted to ensure that we developed guiding principles for reporting that could be readily adopted across a spectrum of organizations, and that they would be sufficiently detailed to be useful, while being general enough to be broadly applicable. In this work, we have focused primarily on comprehensive reports describing complete population PK analyses, rather than memo-style reports or publications.

### Objectives

The objectives of this work were to:

1. Develop and implement a survey of common population PK analysis and reporting practices among pharmacometrics practitioners,
2. Present the survey results to interested stakeholders including industry, consulting and regulatory scientists in order to generate discussion on best reporting practices,
3. Based on this broad consultation, develop a consolidated set of reporting guidelines for population PK analyses.

## Survey description and results

### Description

A single-round electronic survey was conducted in order to elicit information on current views and practices on reporting of population PK analyses. It included 91 questions about different aspects of respondents’ experiences and preferences regarding population PK analyses and reporting. These were organized into five primary sections: (1) respondent experience; (2) purpose and impact of population PK report; (3) components of population PK report; (4) model diagnostics; and (5) modeling practices. The survey mainly contained multiple-choice questions. The survey is available in the online supplemental material. Approximately 3200 members of the MBDD consortium member organizations, including AAPS Pharmacometrics Focus Group (formerly the Population PK, and Modeling and Simulation Focus Groups), ACCP, ASCPT Pharmacometrics and Pharmacokinetics section, and ISoP, were invited to participate in this survey. The survey was available online during the period 6th to 26th November, 2012.

### Survey results

A total of 351 surveys (11 % response rate) were completed and available for analyses. All survey responses were included, whether or not a respondent had completed all questions. Table 1 lists the survey sections and fraction of respondents with complete responses by section.

**Table 1:** Survey sections and fraction of respondents by section

Section	Percentage of respondents
Respondent characteristics	99
Respondent experience and use of reporting	99
Report components and importance	75
Model diagnostics	51
Modeling practice	63–78
General comments	21

### Respondent characteristics

The respondents were generally experienced in population PK analysis, with 87 % having at least 5 years post graduate experience in pharmaceutical science, and with 72 % considering their knowledge in population PK analyses at least intermediate (Table 2). Sixty percent (60 %) of respondents had personally performed, and 81 % had reviewed population PK analyses in detail in the 2 years prior to the survey. The survey did not interrogate the type of organization in which the respondents worked, i.e. industry, regulatory or academic, etc.

**Table 2:** Respondents’ experience and knowledge with population PK analyses

Question	Category	Percentage
Years of post-graduate experience?	1–4 years	11
	5–9 years	18
	10–19 years	34
	20–29 years	20
	30+ years	15
Knowledge of population PK reports?	Not knowledgeable	2
	Basic	26
	Intermediate	31
	Advanced	27
	Expert	14

### Report audience and use

When asked about the main audience of population PK reports, 44 % of respondents answered that regulatory reviewers comprised the main audience for these reports (Table 3), while 38 % indicated the main audience as internal technical experts (pharmacometrics, PK, clinical pharmacology, biostatistics). Internal non-technical experts including clinical, regulatory and governance were considered by only 15 % of respondents as the main audience. The results were consistent regardless of the respondents’ experience or knowledge in population PK analyses.

**Table 3:** Perceived audience and purpose for population PK reports

Question	Category	Percentage
Audience of population PK reports?	Regulatory	44
	Internal technical	38
	Internal non-technical	15
	Other	3
Main purpose of population PK analyses?	Covariate effects	52
	Data integration	25
	PK documents	14
	Other	10
Major impact of population PK analyses	Dose selection	57
	Support PK/PD analysis	31
	Regulatory checkbox	8
	Other	4

Fifty-two percent (52 %) of respondents indicated testing and identification of covariate effects or effects in special populations as the most important purpose of population PK reports, followed by integration of PK information across clinical trials (25 %) (Table 3). Formulation of dosing recommendations for clinical trials or labeling (57 %) and ground work for exposure–response analysis (30 %) were the two most important impacts of population PK reports identified by respondents.

### Report components

A list of 37 common content elements (including main sections, subsections and other frequently reported analysis elements) found in a typical population PK report was queried in the survey to identify respondents’ preferred location and perceived importance in a report. The options for preferred location were “Report Body”, “Combine with Another Section”, or “Appendix”. Approximately 75 % of the respondents provided a response in this section. Respondents were also asked to rate the importance of a given element on a 1–5 scale, with 5 being “Extremely important” and 1 being “Not Very Important”. A final question queried the preferred audience for each section. We report on the preferred location and importance questions here (full survey results are available upon request from the authors). For each content element, a weighted importance was calculated as the sum of the product of the numeric response value by the percentage of respondents giving a particular response, i.e.

$$Weighted \;Importance = \mathop \sum \limits_{1}^{{n_{level} }} \left( {r \times P_{r} } \right)/n_{level}$$

where *n*_*level*_ is the number of possible response levels, *r* is the response value (1–5), and *P*_*r*_ is the percentage of respondents giving that response.

**Fig. 1:**
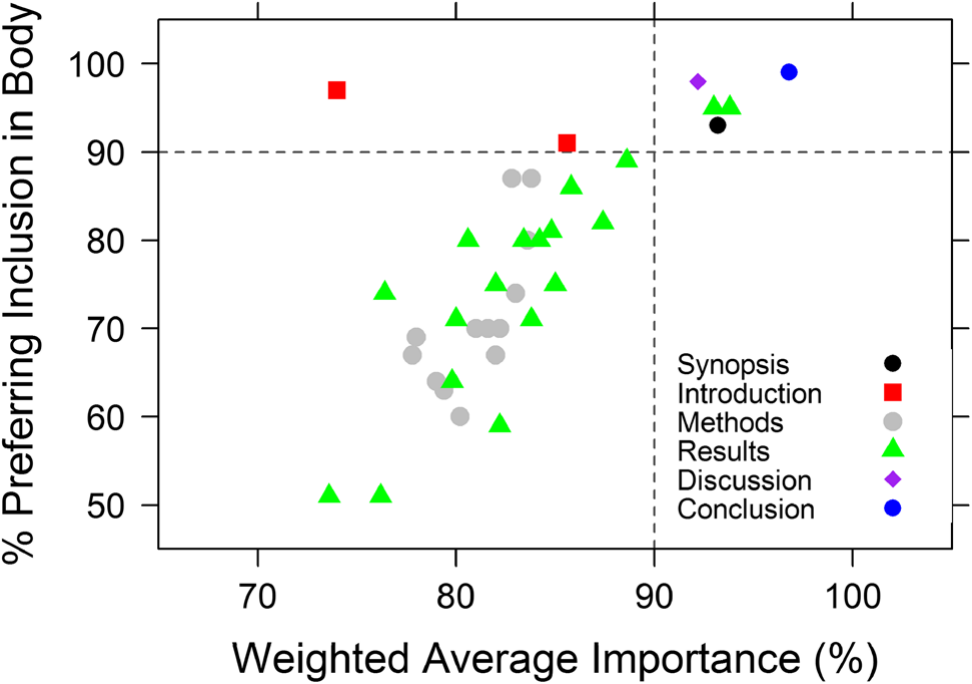
Components of a population PK report and their perceived importance. Each *symbol* represents the response for a single report section, subsection or content element

As shown in Fig. 1, there was a clear correlation between a desire to include a section or element in the body of the report and the perceived importance of the section. The exception was for introductory material, with over 95 % respondents preferring to see the Introduction included in the report body, but assigning relatively little importance to this section. The elements that >90 % of respondents preferred to be included in the report body and which had perceived weighted importance >90 % included the Synopsis, Conclusions, Discussion along with Final Model description, and Application/Interpretation of Model Results, both within the “Results section”.

**Fig. 2:**
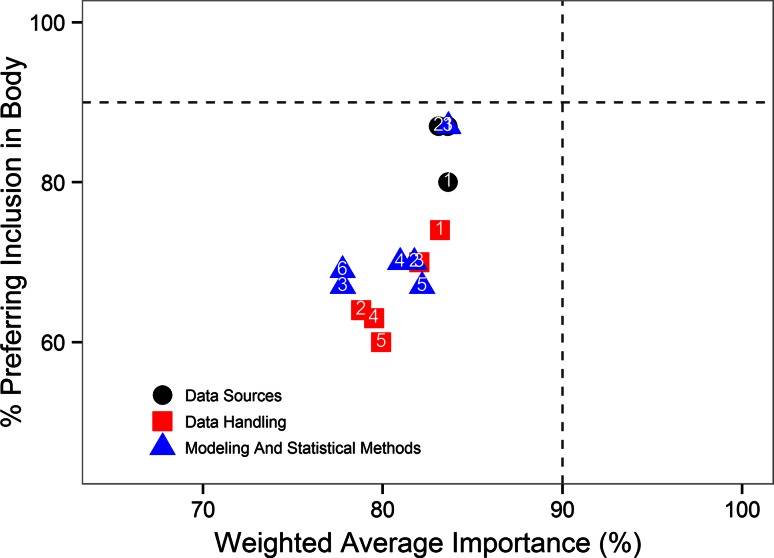
Preferred location and importance of content elements within “Methods section”: *Data Sources* (*circles*): *1*. overall data sources *2*. study design *3*. population. *Data Handling* (*triangles*): *1*. overall data handling *2*. handling of missing data *3*. handling of covariates *4*. handling of outliers *5*. data exclusions. *Modeling and Statistical Methods* (*squares*): *1*. general approach *2*. structural model development *3*. random effects *4*. covariate model development *5*. model qualification *6*. simulation methods. Note: X-axis jitter added to data to distinguish overlapping values

None of the content elements in the “Methods section” had weighted perceived importance over 85 % or had over 90 % of respondents preferring to see a given element in the report body (Fig. 2). The three Methods elements with over 80 % of respondents preferring to see them in the report body were the “General Modeling Approach”, “Study Design” and “Population Description”.

**Fig. 3:**
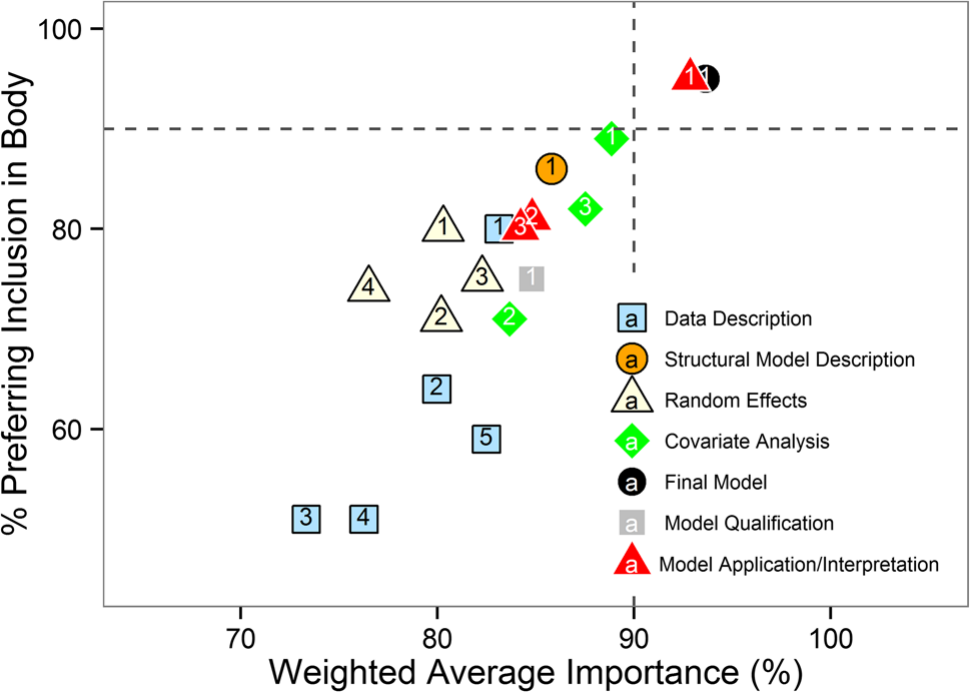
Preferred location and importance of Results content elements: *Data Description* (*light blue square*): *1*. demographics *2*. covariate distributions *3*. sampling time distribution *4*. display of raw data versus time *5*. other. *Structural Model Description* (*orange circle*): *1*. overall random effects (*light yellow triangle*): *1*. overall *2*. residual variability *3*. inter-individual variability *4*. inter-occasion variability. *Covariate Analysis* (*green diamond*): *1*. overall *2*. covariates tested *3*. covariates selected. *Final Model* (*black square*): *1*. overall. *Model Qualification* (*gray circle*): *1*. overall. *Application/Interpretation of Model Results* (*red triangle*): *1*. overall *2*. simulation results *3*. size of identified differences among covariates. Note: X-axis jitter added to data to distinguish overlapping values

Within the “Results section” (Fig. 3), the overall “Covariate Analysis” and “Structural Model Description” each had perceived importance >85 % and over 85 % of respondents preferring inclusion in the report body (in addition to the “Final Model Description” and “Application/Interpretation of the Results”, referred to above).

These results suggest that respondents found the most value in overall descriptions of the analysis, methods and results with particular importance being placed on higher level content elements. Overall, based on these data and information from the survey comments, the population PK report is perceived as a communication tool summarizing the general methodology and study data while focusing on the final model and its clinical application.

### Model diagnostics and modeling practice

The next two survey sections addressed questions related to what supporting analysis should be included in order for the report to be considered a credible, well-qualified document supporting the analysis recommendations. Survey respondents were asked to rate the utility of different model diagnostics and were queried about specific practices they employ in developing a population PK model.

Similar to the survey section on content elements, respondents were asked their preferred location for different model diagnostics, and to rate the importance of each diagnostic. Additionally, respondents were asked how frequently a given diagnostic plot or analysis should be included in the report. Possible responses were “always”, “optional”, or “in special cases”. Response results were divided into three categories: generally included: defined as those that >80 % of respondents would include in the report body and with >80 % weighted importance; intermediate inclusion: those with 70–80 % weighted importance and inclusion, and as needed: those diagnostics with <70 % weighted importance or that <70 % of respondents indicated they always include in the report body (see Table 4).

**Table 4:** Model diagnostics versus inclusion category

Generally included	Intermediate inclusion	As needed
Precision of estimates	PRED vs. DV	Shrinkage
Comparison of model results to observations	IPRED vs. DV	Bootstrap
Magnitude of residual variability	CWRES vs. Time	Random effects distributions
Magnitude of interindividual variability	CWRES vs. PRED	OMEGA matrix
Visual predictive check	Model predictions and observed data vs. time	Histogram of Etas
Traditional PK summary parameters		Case-deletion
Parameter values vs. covariates of interest		Model development trail

Generally, respondents confirmed that model diagnostics are an important component of any population PK report. There is also some overlap in the diagnostic analyses that were queried; for example, a visual predictive check (VPC) is a type of comparison of the model results to observations [9]. As shown in Table 4, standard diagnostics related to precision of parameter estimates, estimates of inter-subject and residual variability, and direct comparison of model predictions to observed data were in the generally included category. Diagnostics, such as conditional weighted residuals versus population predicted value (PRED) or shrinkage, that could be considered more closely related to statistical performance of the model were in the two categories that might be less frequently included in the report body.

### Modeling practice

To elicit participants’ attitude towards modeling practice, we asked nine questions that potentially could influence acceptance, perceived beliefs, and usage of certain modeling methodologies. A total of 63 % of respondents answered at least one of the nine questions in this section of the survey. Most of the respondents who did not answer questions in this section described themselves as having basic or no knowledge of population PK analyses. Each question in this section queried respondents’ degree of acceptance of different PK modeling practices. Possible responses to the questions were (1) “Of course!”, (2) “Usually”, (3) “Sometimes, with justification”, and (4) “Never”.

The majority of respondents, 60 %, indicated that it is acceptable, “sometimes with justification”, to include a covariate effect on parameters with no random effect. Similarly, 53 % indicated that it is occasionally acceptable to add a random effect based on goodness-of-fit or objective function value “with justification”.

Fifty-nine percent (59 %) of respondents indicated that it is sometimes acceptable to report a final model that did not successfully estimate the model variance–covariance matrix (i.e. completion of the $COV step in NONMEM), “with justification”, while 65 % would sometimes accept the use of first order (FO) estimation. Simulation-based estimation methods were considered acceptable by 42 % of respondents, again, with appropriate justification. Respondents reported using a range of simulation-based estimations methods; however SAEM was accepted by the majority of respondents as an acceptable simulation-based estimation method.

To put covariate analysis into context, we asked if covariates could be excluded due to large shrinkage, very small effect size, or lack of precision. Forty-seven percent (47 %) reported “usually” excluding covariate effects due to large shrinkage, with 42 % excluding covariate effects for very small effect size, and 44 %, removing covariates from the model because of lack of precision in the parameter estimates.

The final question referred to the preferred method for handling BLOQ values, specifically whether the respondent prefers to employ the so-called M3 method, which maximizes the likelihood considering measurable values together with the likelihood of observations being BLOQ [10]. Forty-four percent (44 %) of respondents reported using the M3 method to handle values that are BLOQ. The key finding in this section was that the survey respondents found many methodological variations acceptable as long as their use is adequately justified.

## General recommendations

### Key questions to address

The dual objectives of a population PK report are (1) to communicate key findings and recommendations to stakeholders (e.g. internal stakeholders or regulatory agencies); and (2) to make the analyses reproducible by summarizing the methods, data used, and results obtained in the analysis. To achieve that impact, the population PK report should address the following questions:

- Why do the analysis?
- What information did you use?
- How did you do it?
- What did you find out and why does it matter?
- Is the model good enough?
- What are the key assumptions or limitations to the analysis interpretation?

### Why do the analysis?

It is critical to obtain alignment of objectives with key internal stakeholders before embarking on a population PK analysis, and it is important that the analysis objectives be spelled out in the population PK report. A data/modeling analysis plan (DAP) can be a useful tool to prospectively facilitate such alignment, provided care is taken to spell out the key strategic questions the analysis is intended to address, in addition to describing the planned technical conduct of the analysis.

### What information did you use?

A detailed description of the data used for the analysis is important in order to assess whether the dataset is adequate to support the intended purpose of the analysis and whether certain limitations can be identified a priori. For example, identification of the influence of a particular covariate may not be supported by an analysis based primarily on small Phase 1 studies. Conversely, model estimation of peak plasma concentration or shape of the PK profile is difficult to assess by analysis of sparse, trough concentration samples.

### How did you do it?

This technical portion of the report will describe the general modeling approach, and should also include the relevant specific technical details used in conducting the analysis.

### What did you find out and why does it matter?

The report needs to highlight the main findings and relate these results to the initial analysis objectives. Further, the report needs to place the results in the overall context of prior knowledge about the compound and the intended patient population. Clear, concise descriptions of the key findings, analysis context, and analysis strengths and weaknesses will allow informed decisions to be made on the basis of the analysis.

### Is the model good enough?

The report should include demonstration that the model describes the data adequately and is sufficiently detailed to be fit for the purpose outlined in the analysis objectives. Rationale for modeling assumptions and choices should be described to allow the reader to assess whether the conclusions are robust enough to support recommendations based on the results.

### What are the key assumptions or limitations to the analysis interpretation?

No model completely describes all aspects of a given dataset. The strengths and weaknesses of the analysis and possible limitations of the underlying data should be discussed to increase the confidence in the conclusions and help the readers assess the degree to which the findings reflect true characteristics of the target population or are dependent on model assumptions or data limitations.

### Report audiences

The survey results suggested that the main audiences for population PK reports were regulatory reviewers and internal technical experts, two groups that are generally technically adept. However, in the authors’ experience, there is a significant fraction of internal and external stakeholders who do not have specific technical expertise in pharmacometrics, but who often play an important role in reviewing the reports and in making decisions based on the analysis results.

To maximize the impact of population PK analysis, key findings need to be reported in such a manner that a majority of informed stakeholders can understand the major findings and feel confident, with the participation of pharmacometricians, in making decisions based on the analysis. Thus, the population PK report should include portions that are meant to be accessible to all readers. These sections should include description of the problem to be addressed and the key analysis findings, each expressed in language that a scientifically literate audience would be able to understand. This means, for example, that covariate effects would be described in terms of their impact on concentration or measures of overall drug exposure, rather than their impact on specific clearance or volume parameter values. These report sections could be described as being primarily decision-focused.

It is also clear that the population PK report serves a critical purpose in facilitating technical review of the work and in documenting the analysis for future reference. In this role, the report needs to serve as a repository for the technical details regarding the methodology, model qualification, and detailed analysis results. These analysis documentation sections should be written so that a technically adept reader can comprehensively understand the analysis methodology and detailed results.

In summary, there are two key audiences for population PK reports: (1) well-informed but non-technical readers whose interest is in the main analysis results, its ramifications, and the ability of the analysis to support key drug development decisions, and (2) technically adept readers whose task is to review the analysis for technical soundness or who may be responsible for repeating or expanding the analysis at a later time. Different sections of the report should be written to address one or both of these audiences.

### Role of data or modeling analysis plan

Population PK analyses are focused on estimation of key parameter values, variability between subjects, and covariate effects on exposure. An important concern is the degree of certainty with which these characteristics can be credibly assessed from a given dataset and analysis methodology. A DAP is often prepared in order to prospectively describe the data, methods and analysis objectives and to help foster acceptance of the planned population PK analysis by a drug development team. If a DAP has been prepared, the DAP should be seen as a useful planning and communication tool, but cannot precisely prespecify a particular method of analysis or model-building path. For analyses that are carried out over an extended period of time or that are updated as data become available, the DAP may be seen as a living document. Thus, there may not be a need to exhaustively address deviations from the plan in the body of the population PK report. However, it is recommended that one critically assess the need for deviations from the plan, and it is often valuable to the reader to understand what was learned in the current analysis that may have caused a given deviation.

Important elements to include in a DAP are the purpose for performing the analyses, key questions/applications the analysis will address, prior information from e.g. previous trials, compounds and/or literature, choice of data, lists of covariates to be examined and general methodologies, as well as specific assumptions and limitations.

## Guidelines for report sections

### Overall report structure

In considering the overall report structure, it was deemed important to have the overall structure be consistent with current practices in general scientific reporting and with other technical reports that are found in regulatory submissions. We suggest that the population PK report include the following main sections:

I. Synopsis
II. Introduction
III. Data
IV. Methods
V. Results
VI. Discussion
VII. Conclusions
VIII. Appendix (if needed)

This structure is similar to the overall structure recommended in previous population PK regulatory guidance [3, 5]. Recommended guiding principles for each of these report sections are detailed below.

### Synopsis section

- Audience: all readers
- Focus area: objectives and impact of analysis

The Synopsis is arguably the most important section in the population PK report. The Synopsis should be a stand-alone section that states the recommendations, and summarizes the evidence supporting them in clear language that can be understood by all stakeholders. If the analysis results in a labelling change, portions of the synopsis might be included in the Summary of Clinical Pharmacology as part of an NDA review. This section should highlight the objectives of the analysis and provide a high level summary of the data and the methodology utilized. Examples of key results might include a list of covariates tested and identification of those that have meaningful impact on the PK of a drug, or if dosage adjustments are warranted in particular populations. Results displayed in this section should be shown or described in terms of the impact on drug concentration or exposure, and not in terms of specific PK parameters, in keeping with the goal of communicating the analysis results and impact to a generally informed, but not necessarily technically inclined audience. In addition, important summary graphs or tables that support decision making could be incorporated in this section, for example, forest plots depicting the impact of covariates on C_max_ or AUC. This section should be reasonably succinct, and not a lengthy repetition of material found in the body of the report. For a typical analysis, this section might be approximately two pages long, though, obviously, the scope and complexity of the material to be presented will dictate the length of this or any other section.

### Introduction section

- Audience: all readers
- Focus area: background and motivation for the analysis

Similar to the Synopsis, the Introduction is generally targeted for all audiences and should therefore contain pertinent information that provides context and motivation for the analysis. This section should provide sufficient background information on the pharmacology of the compound, the target indication and the PK characteristics based on historical information and the stage of drug development to motivate the analysis and approach. A clear statement of the analysis objectives is an essential component of this section. Typically, this information would be summarized in a single page.

### Data section

- Audience: technical readers
- Focus area: data sources, relevant aspects of study design, description of data handling and issues

In this section, a description of the study data and the processes used to generate the analysis data set should be given.

The data upon which the analysis is based is a key determinant of whether the results can support the intended objectives. Therefore, it is critical that a description of the data used in the analysis along with a description of any modifications or derived quantities be included in the report. This section would normally include a short description of the study or studies that generated analysis data, with special importance attached to the number of subjects, number and timing of samples per subject, disease status in each study, and any pertinent demographic or laboratory data. The data summarized in this section should be consistent with the stated analysis objectives. Typically, approximately two paragraphs per protocol would be sufficient to summarize pertinent study data. It is also quite useful to show this information in tabular form.

Specific details regarding, for example, rules for removal of data with missing information, imputation of missing covariate information, removal of outliers, etc. might be provided in the body of the report together with the rationale for specific decisions made during the creation of the datasets. Listings of specific data points removed from the dataset, along with the reason for omission would normally be included in an appendix. If datasets are modified over the course of the analysis, the file name of each version of the dataset must be unique and provided in the appendix. This allows verification of the different datasets utilized for the various model files.

Table 5 provides guidelines with respect to the data elements that are considered to be essential in the main body of the report versus those that could be placed in an appendix in supplementary material. If a DAP exists, that document may be referenced to avoid redundancies in the report while highlighting only the deviations from the proposed analyses in the main body of the report.

**Table 5:** Guidelines for placement of data elements

Generally included in main body of report	May or may not be included in report body	Usually included in appendix
Description of studies in source dataset	Methods for covariate imputation	Excluded data and the reasons for exclusion
Study design and study population		File name of each version of the dataset and the modifications made
Sampling strategy, number of subjects		
Table of demographic and covariate information		
Handling of missing data/imputation methods for missing PK data		
Handling of outliers		

### Methods section

- Audience: technical readers
- Focus area: technical methods with focus on the most important aspects, balanced with other details to be included in Appendix

Consistent with the purpose of any scientific communication, a population PK report needs to provide sufficient detail of the modeling exercise to allow reproduction of the analysis. However, most methodological detail would normally be of interest to a primarily technical audience, and certain methodologies may be considered sufficiently standardized to be described in an appendix, or to be included by reference (Fig. 2). Dependent on whether a DAP exists, some of the elements could be referenced to the DAP (as a stand-alone document or as an appendix to the report) rather than being repeated in the report body. If a DAP does not exist, standard technical details (e.g., construction of the structural model, standard methods for covariate search) could be included in an appendix while the estimation methods specific to the analysis should be included in the body of the report. Table 6 provides guidelines with respect to the elements of the method section that are considered to be essential in the main body of the report versus being embedded in an appendix.

**Table 6:** Guidelines for placement of methods subsections

Generally included in main body of report	May or may not be included in main body of report	Usually included in appendix
Model structure, including both a diagram and equations	Construction of structural model	DAP
Prior knowledge about covariate effects	Identification of random effects	Description of assay methods or reference to appropriate documentation
Software, fitting algorithm	Handling of missing data during model development	
Covariates to be examined	Sensitivity analyses (e.g. impact of outliers)	
Covariate selection methods		
Model performance/validation		
Simulations—methodology, inclusion of uncertainty		
Statement of lower limit of assay quantitation		

### Results section

- Audience: technical readers
- Focus area: final model description, diagnostics and qualification

The results section targets a technical audience and details the specific outcomes and applications of the analysis. Recommended elements of the results section include:

- Model development table for key models, including structural models and covariate evaluations
- Reasoning for selection of key models at each stage of the model development process
- Form of the best selected model
- Final parameter estimates, including uncertainty (SE)
- Model performance/qualification
- Simulation or other model applications, e.g., impact of significant covariates on PK parameters, exposure and/or dose selection

A description of the best selected model and the application of the model to address the project objectives should normally be included in the body of the report. This recommendation is consistent with the survey results (Fig. 3). Key model qualification plots (see examples in [5]) should also be presented here. The most critical element of the results section is the interpretation and/or application of the model. Supporting tables or figures that address the objectives and enhance communication of the results of the analysis are essential. Figures should be clearly labelled with text and symbols large enough to be clearly legible. Judicious use of color can greatly aid in distinguishing different plotted elements. Forest plots (see for example [8]), density distributions or histograms may be useful tools to visualize the results and enhance their interpretation.

Depending on the purpose of the report and to improve readability for non-technical audiences, other elements may be included in the body of the report or in one or more appendices. For example, if the primary purpose of the report is to inform dose selection, this should be the major focus of the results in the body of the report and all supporting model development results may be consolidated in an appendix.

A description of the development pathway needs to appear somewhere in the popPK report. It may be appropriate to place an abbreviated table describing only the key models examined in the report body, with the detailed modeling table placed in the appendix. Obviously, placement of model development results in an appendix does not lessen the importance of a rigorous model selection and qualification process. The rationale for model selection must always be clearly presented and the model must be adequately qualified per current guidelines (FDA/EMA guidelines) and state-of-the-art methodologies. Table 7 provides guidelines for placement of key elements of the results section.

**Table 7:** Guidelines for placement of results subsections

Generally included in main body of report	May or may not be included in main body of report	Usually included in appendix
Equations describing the form of the best selected model	Description of final analysis dataset(s)	Detailed, comprehensive model development table(s)
Final parameter estimates	Reasoning for model selection—Model development table for key models	
Key model qualification plots (e.g., DV vs PRED and IPRED, VPC)	Model qualification	
Tables and/or figures illustrating simulation results or other model applications		

### Discussion section

- Audience: all readers
- Focus area: results interpretation, physiologic and mechanistic context

A discussion of the analysis results is an integral component of a population PK report and is intended for both technical and non-technical readers. It should be presented in plain language that is understandable to all stakeholders, non-technical as well as experienced clinicians and pharmacometricians. The discussion is not intended to simply restate the important results, rather, its purpose is to interpret the modeling results and explain their clinical relevance in the context of prior knowledge and with an emphasis on how the results address the project objectives. The discussion may be challenging for report authors, since this is the main section where the impact of the technically-focused “Results section” is explained and placed in context.

The Discussion should include comment on each of the questions listed in “General recommendations” section. Recommended elements of the discussion include:

- Summary of principal findings, e.g., impact of covariates on pharmacokinetics
- Explanation of the relevance of modeling (technical) results, for example, description of the influence of covariates on exposure, safety and efficacy and, in turn, on dose selection or adjustments for specific patient subgroups
- Interpretation of the results in the context of prior knowledge about the drug or other drugs within the same class of compounds
- The robustness of the findings considering the assumptions and identified limitations of the data, model and method with discussion of caveats

### Conclusions section

- Audience: all readers
- Focus area: impact of findings

The conclusion is a succinct summary of the major findings of the analysis and their relevance, and should be written in language that can be understood by a nontechnical audience. The conclusion may be presented as a single summative paragraph or a bullet list.

## Regulatory considerations

Results from population PK analysis submitted to the regulatory agency need to be accompanied with a structured population PK report. FDA does not have any specific recommendations regarding the length of the individual sections of the population PK report, use of a particular format, or inclusion of specific graphs for population PK reporting. The intent of this section is not to be prescriptive but to provide general expectations and considerations regarding population PK reporting from a regulatory perspective and also to highlight the potential advantages that standardization may offer.

Population PK analysis and associated reports play an important role during regulatory review. The report serves as a primary guide for the reviewers to formulate regulatory decisions based on population PK modelling. The description of the sponsor’s model and methodology help identify parts of the analysis that need to be reproduced for confirmation or which may need to be further developed to ensure a complete review of the PK data. The report also serves as a source document to providing relevant figures (model diagnostics, forest plots relevant to the labeling decisions etc.) and tables (description of studies, final parameter estimates etc.) that may be included in regulatory review documents.

Therefore, it is important and usually in the sponsor’s best interest that the above information be readily available to reviewers in order to assist their efficient review. It must also be recognized that review cycles are frequently short and population PK analysis is just one of several parts of a submission falling in the pharmacometrics reviewer’s scope of responsibility. The sections of the proposed annotated label that contain information based on population PK analysis should provide hyperlinks to the relevant population PK report. In certain submissions, for example those addressing pediatric indications, population PK analysis along with exposure–response analysis might be a central element of the review. Currently, there is substantial variation in report structure, location of contents and details within these reports. The objectives of this working group are well-aligned with those of pharmacometric scientists in regulatory agencies in facilitating the efficient and effective review of population PK analyses.

For regulatory review, it is important that the report synopsis is focused on the key decisions and variables of interest. For example, if there is a dosing recommendation for a specific population based on population PK, it is useful to know the impact of the specific covariate on AUC and/or C_max_ (if applicable), rather than reporting only the effects on a specific PK parameter such as CL or V_d_. There is a need and value to be gained in making these reports accessible to an interdisciplinary audience. If the recommendations outlined in this article are followed, it is hoped that reviewers from other disciplines may be able to better understand the rationale behind labelling recommendations based on population PK analyses.

The review of the population PK report submitted by the sponsor is an integral part of the pharmacometric review process. The synopsis is one of the critical components for FDA reviewers, providing a high level summary of the population PK analysis and the recommendations based on the population PK modelling. In addition, the population PK report is usually the source of the base and final model control streams and outputs. Therefore, it is important that the model outputs included in the report match the model output generated when the actual model is run by the reviewer using the sponsor’s model code and dataset. The population PK report is also an important vehicle to understand the technical aspects of the population PK modeling process including model development, model evaluation, simulation etc. Moreover, key tables and figures from the sponsor’s report may be included by regulatory reviewers when describing sponsor’s analysis in the pharmacometric review.

Summarized below are some of the considerations for key sections when reporting population PK results to the regulatory agencies. Specific suggestions that may facilitate the review process include the following for each section:

- Synopsis: This is the most important stand-alone section of the population PK report summarizing the objectives, data, methodology and recommendations. The target audience of this section may be people with minimal or no hands-on experience with population PK analysis. It is important to present the results in terms of effects on drug exposure (e.g. AUC, C_min_, C_max_, C_avg_) and not PK parameters when describing the impact of covariates on the pharmacokinetics. The synopsis should also include a brief justification on why the available data is adequate to evaluate difference in exposures in specific populations.
- Data section: It is recommended that the distinction be made between available data and final data used for model building and evaluation. Tabulated summaries of utilized studies should be included summarizing number of patients, PK samples, demographic characteristics etc. The lower limit of quantification should be reported. Furthermore, justification for not including studies with readily available and potentially informative data should be provided. Finally, the approach used for handling of outliers and missing data should be included in this section.
- Methodology: This section is should be sufficiently detailed to allow for replication of results. In addition, key simulations used for labelling recommendations resulting from the analysis should be described in detail. Methods for incorporating variability or parameter uncertainty and deriving confidence intervals, or prediction intervals should be clearly described in the main body of the report.
- Results: Key results of the analysis could also be presented utilizing format of tables or figures for easy readability and interpretation. Apart from the recommended elements as described in the ‘Results’ section above, the following information is also considered helpful to pharmacometric reviewers:
  (i) If the population PK model includes data from dedicated clinical pharmacology studies (e.g. renal impairment, hepatic impairment or drug interaction studies), the results obtained from population PK should be compared to those observed in the dedicated studies. The consistency or inconsistency of the results between these two approaches (typical non-compartmental approach of comparing PK in various groups utilized in the dedicated clinical pharmacology studies versus assessing the impact of a covariate using pooled data via population analysis)
  (ii) In addition to the information on decrease in objective function value by the inclusion of the covariate, the information on “how much variability is explained by inclusion of the covariate?” is also useful and should be included.
  (iii) The variability in parameter estimates between subjects should be reported as %CV while precision of the parameter estimate should be reported as % RSE (% Relative standard error) or 95 % CI.
  (iv) Depending on the objective of the analysis, VPC plots should be stratified by relevant covariates to illustrate the performance of the model in specific subgroups.
  (v) A table comparing the parameter estimates from the base and final model should be included for easy side by side comparison.
  Furthermore, in accordance to what has been discussed above, the use of innovative and informative visual representation of results, that may include color, may facilitate understanding the clinical implications of the analysis is encouraged.
- Discussion: Apart from the recommended elements as described in the Discussion section above, it is also important to discuss the adequacy or inadequacy (if it exists) of the data to support recommendations based on the model.
- Appendices: At the minimum, the model code and outputs for the base and the final model should be included. The code and outputs of key intermediate models may also be included as deemed necessary. A run record detailing the steps undertaken for the analysis should be presented. Wherever necessary, methodology and codes for generating the key figures other than standard diagnostic and individual plots (e.g. figure describing simulation of an alternate dosing regimen) should be provided. There is no need to reproduce tables of individual data since this information is already available in the analysis dataset submitted.
- Electronic files: Sponsors should refer to the FDA Pharmacometrics website [11] for general guidance on expectations of submitting pharmacometric data and models. It is critical that datasets and model files submitted for the base, final and key intermediate models are the same as those used for generating the model outputs in the appendices of the report. It is also important to include the unique subject identifier information for each subject in the population PK dataset that is same as used in the individual clinical study report datasets. This information is vital if data integration is required between the individual level output (e.g. individual post hoc estimates for clearance or volume of distribution) generated from the population PK model and the efficacy or safety datasets from the individual clinical study reports.

## Conclusions

Standardized reporting is a positive step towards the industrialization of pharmacometrics [2]. A number of publications focus either on the technical aspects [4, 7] or reporting for a specific audience, e.g. preparation of publications involving population PK [8]. In contrast, our focus was to provide general recommendations for reports that serve the dual purpose of providing a repository of technical information and a communication tool for informed stakeholders. The intent was not to be prescriptive, but to provide general recommendations, and thus to highlight the potential advantages that standardization may offer rather than to provide an exhaustive description of technical details.

Initially, a survey was implemented to elicit common PK reporting and analysis practices among practitioners. The survey results were used as a basis for consultation and collaboration among practitioners from a wide experience base, including representatives from industry, regulators and consultants providing population PK analysis services. Derived recommendations are general in nature and can be utilized by different types of organizations, e.g. academic, clinical, industry, or regulatory.

The benefits of this standardization for reviewers, including regulators, are as follows:

- Clear objectives are always stated
- Consistency in terminology, figures and content permits efficient review,
- Relevant, easy to find content increases efficiency in interpretation,
- Discussion of previous findings puts the analysis findings into a broader context.
- The synopsis focuses on major findings of interest and technical detail is balanced with a decision/recommendation focus,
- A well-written synopsis provides non-technical audience with an understanding of the application of population PK analysis.

## Electronic supplementary material

Supplementary material 1 (PDF 108 kb)

## Footnotes

1 Views expressed in this manuscript reflect the opinion of the individual contributors, and do not reflect the position of the U.S. Food and Drug Administration.
