# Supplementary material for: Reporting guidelines for population pharmacokinetic analyses
Source: J Pharmacokinet Pharmacodyn. 2015 Apr 30;42(3):301–14. doi: 10.1007/s10928-015-9417-1 (PMC4432104; doi:10.1007/s10928-015-9417-1)
Supplement: Supplementary file 1 — Supplementary material 1 (PDF 108 kb) [file 10928_2015_9417_MOESM1_ESM.pdf]

2012 PopPK Reporting Survey

This survey aims to inform the work of the Model-Based Drug Development (MBDD) Consortium PopPK Reporting Taskforce as it works to create standards for the reporting of Population Pharmacokinetics analyses. Your input is very much appreciated.

Questions are spread over a number of pages. You can advance by clicking NEXT>>, revisit prior pages by clicking <<PREVIOUS, or <<PAUSE>> the survey and return later. Once you reach the final page and have answered all items, click SUBMIT to record your responses.

Thank you for this valuable assistance to our profession.

About you

V0001

1. Roughly how many years of post-graduate experience in pharmaceutical science do you have?

- ○ none (still a student)
- ○ 1 - 4 years
- ○ 5 - 9 years
- ○ 10 - 19 years
- ○ 20 - 29 years
- ○ 30 years or more

V0002

2. How do you rate your knowledge of Population Pharmacokinetics (PopPK) analyses?

- ○ not knowledgeable
- ○ basic
- ○ intermediate
- ○ advanced
- ○ expert

V0003

3a. In the last 2 years, approximately how many PopPK analyses have you *personally performed*?

- ○ none
- ○ 1 - 2
- ○ 3 - 4
- ○ 5 - 9
- ○ 10 - 19
- ○ 20 or more

V0004

3b. In the last 2 years, approximately how many PopPK analyses done by others have you *reviewed in detail*?

- ○ none
- ○ 1 - 2
- ○ 3 - 4
- ○ 5 - 9
- ○ 10 - 19
- ○ 20 or more

<< PAUSE >>	NEXT >>

Reporting use

V0005,V0006

4. In your experience, who is generally the *main audience* for most PopPK reports?
please select the one best option

- ○ regulatory reviewers
- ○ internal technical experts (pharmacometrics, PK, clinical pharmacy, biostats)
- ○ internal non-technical experts (clinical, regulatory, governance)
- ○ other *(please specify)*: ____

V0007,V0008

5. In your experience, what is generally the *most important purpose* of PopPK reports?
please select the one best option

- ○ document major PK characteristics
- ○ test/identify covariate effects or effects in special populations
- ○ integrate PK information across trials
- ○ other *(please specify)*: ____

V0009,V0010

6. In your experience, what is generally the *most important impact* of PopPK reports?
please select the one best option

- ○ inform product dosing recommendations for clinical trials or labeling
- ○ regulatory box check
- ○ ground work for exposure response analysis
- ○ other *(please specify)*: ____

V0011

7. What do you believe are the key decisions that are influenced or impacted by PopPK analyses?

<< PREVIOUS	<< PAUSE >>	NEXT >>

PopPK report components

8. Listed below are possible sections, subsections, and sub-subsections of a PopPK report. Please indicate for each:

a. Where in the report does it belong?
For those you mark "combine", please explain in the comment box at end of question.

b. How important is it?

c. Who is the primary intended audience?

please select one response in each column for each row

V0201:V0237<br>a.<br>where does it belong?	V0301:V0337<br>b.<br>how important?	V0401:V0437<br>c.<br>primary audience?	*roll your mouse over the item to see a description*
?	?	?	**Synopsis**
	?	?	**Introduction**
	?	?	Rationale for Analysis
			**Methods**
?	?	?	Data Sources
?	?	?	Study Design
?	?	?	Population
	?	?	Data Handling
	?	?	Handling of Missing Data
	?	?	Handling of Covariates
	?	?	Handling of Outliers
	?	?	Data Exclusions
			Modeling and Statistical Methods
?	?	?	General Approach
?	?	?	Structural Model Development
?	?	?	Random Effects
?	?	?	Covariate Model Development
?	?	?	Model Qualification
?	?	?	Simulation Methods

Select options:

a. where does it belong?
- report body
- combine with another section
- appendix

b. how important?
- 5 - extremely important
- 4
- 3
- 2
- 1 - not very important

c. primary audience?
- pharmacometrician
- clinician
- biostatistician
- decision maker (internal or regulatory)
- all of the above

a. where does it belong?	b. how important?	c. primary audience?	*roll your mouse over the item to see a description*
			**Results**
			Data Description
?	?	?	Demographics
?	?	?	Covariate Distributions
?	?	?	Sampling Time Distribution
?	?	?	Display of Raw Data vs. Time
?	?	?	Other: __________________
	?	?	Structural Model Description
?	?	?	Random Effects
?	?	?	Residual Variability
?	?	?	Inter-Individual Variability
?	?	?	Inter-Occasion Variability

a. where does it belong?	b. how important?	c. primary audience?	*roll your mouse over the item to see a description*
			**Results** (continued)
?	?	?	Covariate Analysis
?	?	?	Covariates Tested
?	?	?	Covariates Selected
	?	?	Final Model
?	?	?	Model Qualification
	?	?	Application/Interpretation of Model Results
?	?	?	Simulation Results
	?	?	Size of Identified Differences Among Covariates
?	?	?	**Discussion**
?	?	?	**Conclusions**

V0016

9. If you indicated any sections should be combined, please provide details here.

V0013

10. What possible sections / subsections are missing from the list above (if any)?

V0014,V0015

11. What is your preferred term for the subsection entitled "Model Qualification" above?

please select the one best option

- ○ Model Qualification
- ○ Model Validation
- ○ Model Fitness
- ○ other *(please specify)*: ____

<< PREVIOUS	<< PAUSE >>	NEXT >>

Model diagnostics

12a. For each analysis listed, where in the report does it belong?

12b. If you indicated that it should be included, how important is it?

12c. How frequently should it be included?
For those you mark "in special cases", please explain in the comment box at end of

please select one response in each column for each row

Select options:

a. where does it belong?
- report body
- appendix
- omit

b. how important?
- 5 - extremely important
- 4
- 3
- 2
- 1 - not very important

c. how frequently included?
- always
- optional
- in special cases

V0021:V0039 a. where does it belong?	V0041:V0059 b. how important?	V0061:V0079 c. how frequently included?	
?	?	?	pred vs dv
?	?	?	ipred vs dv
?	?	?	cwres vs time
?	?	?	cwres vs pred
?	?	?	precision of estimates
?	?	?	histogram of etas
?	?	?	magnitude of interindividual variability (SQRT(omega) * 100)
?	?	?	omega matrix
?	?	?	shrinkage
?	?	?	bootstrap
?	?	?	case-deletion
?	?	?	magnitude of residual variability
?	?	?	random effects distributions
?	?	?	comparison of model results to observations
?	?	?	parameters values vs. covariates of interest
?	?	?	traditional PK summaries, e.g. AUC, thalf, etc.
?	?	?	model development trail
?	?	?	model predictions (ipred and pred) and observed (dv) vs. time
?	?	?	visual predictive check

V0080

13. If you indicated any analyses should appear "in special cases", please provide details here.

<< PREVIOUS	<< PAUSE >>	NEXT >>

Modeling practice

V0081:V0089,V0090

14. What is your response to each statement below?
please select one option for each row

of course!	usually	sometimes, with justi-fication	never	
○	○	○	○	Do you accept covariate effects on parameters with no random effect?
○	○	○	○	Do you believe random effects should be added based on OF changes or based on other GoF plots?
○	○	○	○	Do you accept a final model without successful covariance step?
○	○	○	○	Is FO ever an acceptable estimation method?
○	○	○	○	Do you accept more recent, simulation-based estimation methods? Which one(s)? ____
○	○	○	○	Do you exclude covariate parameters due to large shrinkage?
○	○	○	○	Do you exclude covariate parameters due to very small effect size (<10%)?
○	○	○	○	Do you exclude covariate parameters due to lack of precision?
○	○	○	○	Do you use the M3 method to handle LLOQ values?

V0091

15. Anything else you would like the MBDD Consortium PopPK Reporting Taskforce to take into consideration as they develop their recommendations?

Thank you!

Please fill in your email address below to register your response so we can remove you from future reminders to complete this survey. Your reply will be handled in strict confidence; your email address and survey answers will not be used for any other purpose or disclosed to others.

Your email address: ____________

Thank you for your valuable time. Please click the button below to submit your response.

<< PREVIOUS	<< PAUSE >>	SUBMIT

© 2012 Readex Research www.readexresearch.com
